# An Adolescent With a Giant Ovarian Cyst and Hyperandrogenism: Case Report

**DOI:** 10.1155/crie/6652681

**Published:** 2025-01-29

**Authors:** Julio César Moreno-Alfonso, Sara Hernández Martín, Lidia Ayuso González, Alberto Pérez Martínez

**Affiliations:** ^1^Pediatric Surgery Department, Hospital Universitario de Navarra, Pamplona, Spain; ^2^Doctoral School, Universidad Pública de Navarra (UPNA), Pamplona, Spain

**Keywords:** ovarian cysts, ovarian neoplasms, polycystic ovary syndrome

## Abstract

**Objective:** To present a rare diagnosis of polycystic ovary syndrome (PCOS) after initial suspicion of malignancy. PCOS is a common endocrine disorder in adolescence characterized by hyperandrogenism and polycystic ovaries.

**Case Presentation:** A 13-year-old female patient was referred for a giant mass noted on examination for metrorrhagia. She had previously presented for hirsutism and acne, treated independently. A multicystic abdominal tumor measuring 21 × 17 × 9 cm was identified, with a nodular image and negative tumor markers, but elevated testosterone and LH/FSH ratio. Therefore, video-assisted ovarian cystectomy was performed. Finally, the patient was diagnosed with PCOS and began hormonal therapy, with improvement of hyperandrogenism.

**Conclusion:** In adolescents with large ovarian cysts, in addition to ruling out malignant neoplasms, PCOS should be considered as these may have similar clinical and radiological presentations.

## 1. Introduction

Polycystic ovary syndrome (PCOS) is one of the most common endocrine disorders, affecting 8%–13% of women and 3.4%–19.6% of adolescent girls [1]. It is characterized by clinical and/or biochemical evidence of hyperandrogenism, oligo-anovulation, and polycystic ovarian morphology (PCOM), although polycystic ovary morphology by adult standards is common in normal adolescents, therefore there are no definitive criteria to define PCOM on ultrasound in adolescents [2]. Moreover, its presentation can overlap with other gynecological or endocrine conditions, such as ovarian masses or functional cysts [3]. This clinical and diagnostic overlap underscores the importance of a comprehensive differential diagnosis to guide accurate evaluation and management. We present an atypical manifestation of this highly prevalent endocrinological condition in adolescence, highlighting its diagnostic challenges and implications.

## 2. Case Presentation

A 13-year-old female patient was referred to our center for study due to a giant pelvic mass suspected of malignant neoplasm. She presented with scanty and daily persistent metrorrhagia of 1 year of evolution with no history of cyclic menstruation or dysmenorrhea, being diagnosed with anovulatory cycles. Previously and progressively, she had developed hirsutism and severe nodule-cystic acne involving forehead, temporal, malar, interciliary, and chin regions, for which she was treated with retinoids. Due to persistent symptomatology, she consulted her primary care pediatrician who detected a soft and painless palpable abdominal mass from the pubic symphysis to the supraumbilical region. When asked, the family referred to a mild progressive increase in abdominal diameter in the last months. On examination, a deep voice and moderate hirsutism (23 points on the modified Ferriman–Galwey score) were also noticed.

**Figure 1:**
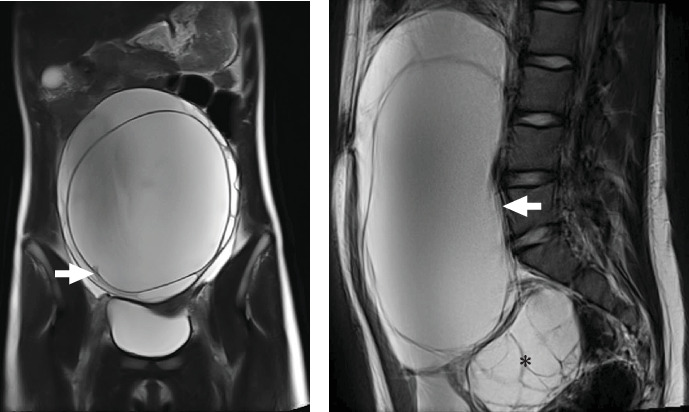
(A) Tumor that involves most of the abdominal cavity and displaces all intraperitoneal structures. It has a 1 cm nodular-papillary projection in its right basal portion with contrast enhancement (arrow). (B) The mass is dependent on the left ovary, is polycystic, and has a large central cavity with thin septa and multiple peripheral cysts (arrow). The right ovary also has multiple cysts (*⁣*^*∗*^).

An ultrasound and an abdominal magnetic resonance imaging (MRI) were performed identifying a large tumor, of 21 × 17 × 9 cm, that occupied the pelvis and most of the abdominal cavity, multicystic in appearance with thin septa and a 1 cm nodular-papillary projection in the right basal portion with contrast enhancement (Figure 1), suggestive of an androgen–secreting sex cord-stromal tumor. This tumor appeared to be dependent on the left ovary and caused compression of the inferior vena cava and a grade II right hydronephrosis. The right ovary also exhibited an enlarged diameter of 7 cm and a multicystic appearance. Tumor markers (CA-125, AFP, b-hCG, and neuronal specific enolase) were negative, with elevated total testosterone and LH/FSH ratio, 2.73 ng/mL and 29.9, respectively. In addition, she presented an altered insulin resistance index (HOMA-IR: 8.4; reference value 1.5–3.2) and hyperinsulinemia (27.4 mU/L).

**Figure 2:**
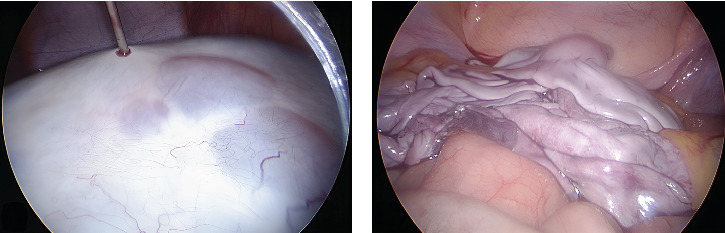
(A) Video-assisted transparietal puncture of the central cyst. (B) Cystic wall after aspiration of its contents sparing the ovarian parenchyma.

All these findings were consistent with PCOS, but the presence of a giant multicystic lesion with solid components, although apparently benign, forced the exclusion of androgen–secreting Sertoli–Leydig tumor or other types of androgenic neoplasms. Therefore, an exploratory laparoscopy was performed, confirming the radiological findings and, after aspiration of 1.7 L of yellow serous intracystic fluid (Figure 2), a video-assisted multiple cystectomy of the left ovary was performed preserving its parenchyma. The right ovary was grossly normal, although with multiple small cysts on its surface. Neither ascites nor adhesion was detected in the abdominal cavity. There were no postoperative complications and the patient was discharged on the second day after surgery. Histopathological study showed ovarian parenchyma displaying multiple cystic follicles, stromal edema, and areas of recent hemorrhage, consistent with polycystic ovary morphology. The patient was diagnosed with PCOS and started hormonal treatment with cyproterone acetate/ethinyl estradiol (2 mg/0.035 mg) single-dose. After 2 months of treatment, the hormone levels normalized (total testosterone 1.94 ng/mL and LH/FSH ratio 0.5), significantly improving the clinical hyperandrogenism and the contralateral ovarian cysts resolved. In the long-term follow-up of over 2 years, the therapeutic response has been maintained, with significant improvement of hirsutism and acne, although with persistence of the deep voice. Ultrasound showed no ovarian cysts and visualization of normal parenchyma on both ovaries, albeit smaller in the left side. The structure of this case report follows the CARE transparent reporting guideline and assent of the patient and consent of parent(s)/guardian(s) was obtained.

## 3. Discussion

PCOS is one of the most common endocrine disorders in adolescents, affecting ~6%–10% of individuals of reproductive age. Its diagnosis during adolescence remains particularly challenging due to the overlap of normal pubertal development and PCOS-related features. According to recent international guidelines, the diagnosis of PCOS in this age group should focus on the presence of clinical and/or biochemical hyperandrogenism and oligo/anovulation, while excluding other etiologies [1–3]. PCOM, a diagnostic criterion in adults, is not considered reliable in adolescents, as multifollicular ovaries are observed in up to 40% of healthy girls 2 years after menarche and are not indicative of pathology [4]. This diagnostic complexity can lead to delays, particularly in cases with atypical presentations, as demonstrated in our patients.

In this case, the presentation of a giant ovarian cyst raised initial concerns for malignancy due to imaging characteristics, including septations and an apparently solid component. Ovarian masses larger than 9 cm with heterogeneous or solid elements, especially when associated with positive tumor markers, carry a malignancy risk of up to 26.5%, further complicating the diagnostic process [5]. Germ cell tumors, such as dysgerminomas and mature teratomas, represent ≈80% of the ovarian neoplasms in pediatrics, with dysgerminomas accounting for ~40% of malignant ovarian tumors in this population [6]. However, sex cord-stromal tumors, particularly Sertoli–Leydig cell tumors, also merit consideration. Although rare, Sertoli–Leydig tumors constitute about 1%–2% of all ovarian neoplasms and are more likely to present with virilizing symptoms due to androgen production [7]. These tumors are known to be large in size and often may exhibit imaging features that overlap with benign and functional ovarian masses, complicating differentiation from other hyperandrogenic conditions such as PCOS.

The heterogeneity in PCOS presentation, encompassing biochemical, radiological, and clinical findings, adds further diagnostic complexity. Hyperandrogenic manifestations in adolescents, such as hirsutism and acne, are common but not specific to PCOS. Additionally, menstrual irregularities and anovulatory cycles, typical during puberty, make it difficult to distinguish normal physiological changes from pathologic features of PCOS. Biochemical hyperandrogenism, often considered a cornerstone of diagnosis, is also challenging to assess due to fluctuating testosterone levels during anovulatory cycles. In this patient, the presence of contralateral ovarian cysts, insulin resistance, and clinical hyperandrogenism supported the eventual diagnosis of PCOS despite the atypical presentation.

Management of PCOS in adolescents requires a comprehensive approach. Hormonal therapies, particularly combined oral contraceptives containing cyproterone acetate or drospirenone, are effective in regulating menstrual cycles and mitigating hyperandrogenic symptoms. Additionally, lifestyle modifications targeting weight reduction are critical for addressing insulin resistance, which is prevalent in this population. Up to 30%–40% of adolescents with PCOS may have metabolic dysfunction, including glucose intolerance or type 2 diabetes, necessitating regular screening for metabolic syndrome [8]. Moreover, mental health comorbidities, such as anxiety and depression, affect nearly 50% of adolescents with PCOS and require integrated psychosocial care [9].

In this case, surgical intervention was required due to the exceptional size of the ovarian cyst and its concerning imaging features. Ovarian-sparing surgery remains the preferred approach in low-risk cases to preserve reproductive potential, particularly in young patients. Intraoperative findings and histological analysis ultimately excluded malignancy, confirming the diagnosis of PCOS-related cysts. It is important to note that while large cysts are uncommon in PCOS, prolonged untreated disease may contribute to their growth. This case highlights the importance of maintaining a broad differential diagnosis when evaluating ovarian masses in adolescents. Although the prevalence of malignancy in pure cystic ovarian lesions is low (<1%), complex, or atypical presentations necessitate thorough evaluation to exclude rare but significant pathologies such as sex cord-stromal tumors. Early recognition and appropriate management of PCOS, even in atypical cases, are essential to improving long-term outcomes and minimizing diagnostic delays.

## 4. Conclusion

In conclusion, this case demonstrates an unusual presentation of PCOS associated with a giant ovarian cyst, emphasizing the diagnostic challenges posed by overlapping features with other gynecologic and endocrine conditions. While PCOS is a common cause of ovarian cysts, cysts of this size are rare and typically associated with neoplastic processes. Accurate diagnosis relies on a combination of clinical, biochemical, and imaging findings, and management should prioritize fertility preservation through ovarian-sparing surgery whenever possible. Early recognition and intervention are crucial for mitigating the long-term metabolic, reproductive, and psychological complications of PCOS in adolescents.

## Data Availability

Data are available upon request due to privacy/ethical restrictions.
